# Ecophysiological responses of *Phragmites australis* populations to a tidal flat gradient in the Yangtze River Estuary, China

**DOI:** 10.3389/fpls.2024.1326345

**Published:** 2024-04-30

**Authors:** Jing Jia, Xiaochao Zhao, Peng Jia, Xin Zhang, Dezhi Li, Yongfeng Liu, Liping Huang

**Affiliations:** ^1^ East China Normal University, Shanghai, China; ^2^ GeneMind Biosciences, Shenzhen, China; ^3^ Foshan University, Foshan, China

**Keywords:** salt tolerance, ferroptosis, *Phragmites australis*, transcriptome, wetland ecosystem

## Abstract

*Phragmites australis* is a prevalent species in the Chongming Dongtan wetland and is capable of thriving in various tidal flat environments, including high salinity habitats. *P. australis* population displays inconsistent ecological performances, highlighting the need to uncover their survival strategies and mechanisms in tidal flats with diverse soil salinities. Upon comparing functional traits of *P. australis* at multiple tidal flats (low, middle, and high) and their responses to soil physicochemical properties, this study aimed to clarify the salt-tolerant strategy of *P. australis* and the corresponding mechanisms. These results showed that leaf characteristics, such as specific leaf area and leaf dry matter content, demonstrated more robust stability to soil salinity than shoot height and dry weight. Furthermore, as salt stress intensified, the activities of superoxide dismutase (SOD), catalase (CAT) and peroxisome (POD) in *P. australis* leaves at low tidal flat exhibited an increased upward trend compared to those at other tidal flats. The molecular mechanism of salt tolerance in *Phragmites australis* across various habitats was investigated using transcriptome sequencing. Weighted correlation network analysis (WGCNA) combined with differentially expressed genes (DEGs) screened out 3 modules closely related to high salt tolerance and identified 105 core genes crucial for high salt tolerance. Further research was carried out on the few degraded populations at low tidal flat, and 25 core genes were identified by combining WGCNA and DEGs. A decrease in the activity of ferroptosis marker gonyautoxin-4 and an increase in the content of Fe^3+^ in the degenerated group were observed, indicating that ferroptosis might participate in degradation. Furthermore, correlation analysis indicated a possible regulatory network between salt tolerance and ferroptosis. In short, this study provided new insights into the salt tolerance mechanism of *P. australis* population along tidal flats.

## Introduction

A coastal wetland refers to the transitional zone between terrestrial and aquatic ecosystems, providing a variety of habitats for many species with unique characteristics of ecosystem composition and structure (Jing et al., 2007; Zhang et al., 2021a). However, due to human activities and global climate change, salt marsh ecosystems worldwide have undergone considerable degradation or complete loss over centuries (Kirwan and Megonigal, 2013; Reijers et al., 2019). Although beach reclamation can augment land development areas and protect against wave or tidal impacts, these dyked marshes may induce undesirable changes in soil physical and chemical properties, such as reducing soil salinity or altering pore water content (Portony, 1999; Feng et al., 2022). These alterations can promote the death or loss of native species diversity and the invasion of non-native species (Warren, 2002).

Wetlands around the world are dynamic ecosystems particularly vulnerable to biological invasion. Due to the particularity of the environment in this area, it is easy to cause a lousy situation of non-native species invasion, so wetland managers should be vigilant. Exotic species pose obvious threats to native species diversity (Wilsey et al., 2015), potentially leading to landscape fragmentation and destroying ecosystem service function (Hulme et al., 2013; Martin et al., 2014). Hence, an urgent need for human intervention is needed, particularly in selecting suitable native foundation species for salt marsh wetland restoration.

Foundation species, often primary producers, provide ecological structures that offer valuable ecosystem services (Angelini et al., 2011; Osland et al., 2022). In the process of restoring degraded ecosystems, foundation species often play a pivotal role in creating habitat structures and sustainable ecosystem development (Krauss and Osland, 2020). Previous studies suggested that identifying and selecting foundation species is a key step in ecosystem restoration (Ellison et al., 2019), necessitating an urgent understanding of enhanced ecosystem services following the planting and establishing of the foundation species (Angelini et al., 2011; Osland et al., 2014). As one of the primary species, *Phragmites australis* (common reed) dominates the ecological community engineering effect in biomass and spontaneous ecosystems (Eller et al., 2020) and has a forward-looking ecological theoretical guidance (Crain and Bertness, 2006; Bonanno and Lo Giudice, 2010) and restoration practice (Hastings et al., 2007). The spatial structure of *P. australis* genotypes under changing environments is linked to patch habitats. It is possible to use habitat patches as test systems to identify suitable genetic resources for ecological restoration in the mosaic landscape (Gao et al., 2012). *P. australis* absorbs nutrients and heavy metals, builds and stabilizes soil, and creates self-sustaining vegetation in urban and industrial areas where many other plants do not flourish (Eller et al., 2020). Wetland managers should consider *P. australis* under a new perspective as a restorative material rather than a non-native invader.

*P. australis* is a cosmopolitan herb with a wide ecological range and is often the dominant species in the ecosystems it inhabits (Clevering and van der Toorn, 2000; Song et al., 2021). Given its excellent stress resistance and crucial role in maintaining the wetland ecosystem structure (Zhou et al., 2021), *P. australis* is an excellent choice for restoration material. Researchers revealed that *P. australis* was remarkably adaptable to abiotic environmental stresses due to its high intraspecific diversity and phenotypic plasticity (Saltonstall, 2007; Guan et al., 2017); thus, it can provide valuable insights into plant responses to adversity. During long-term evolution, *P. australis* populations in diverse habitats have evolved different lineages in ecology, each with its own stable morphological characters and constituting numerous ecological haplotypes (Saltonstall, 2003, 2016). According to previous researchers, only tolerant genotype associated with haplotype O grow under selective pressure in non-riverine habitats (estuaries), whereas more competitive genotype associated with haplotype P dominate in riverine habitats (Lambertini et al., 2020; Liu et al., 2021). Previous research into the haplotypes of *P. australis* populations in eastern China revealed that O and P haplotypes coexisted (An et al., 2012). This divergence has been steadily broken down in recent years, and the haplotype P, representing an octaploid lineage (Liu et al., 2022), is now extensively dispersed in eastern China, with a concentration in coastal salt marshes (Liu et al., 2023).

Soil salinity is a growing global issue that negatively impacts plant growth, development, and crop yield. This problem is exacerbated by natural soil salinity, human practices such as irrigation, and climate change (Hassani et al., 2021). Climate change can increase salinization through rising sea levels or drought-induced evaporation (Talke and Jay, 2020; Hassani et al., 2021). Although the biomass of the plants and the area of spruce trees in the wetland ecosystem decreased during extreme events, the *P. australis* population density remained at the same level (Ojdanic et al., 2023). Its large biomass still plays an important role in the ecological service function of the habitat. Studying how plants adapt to soil salt stress is crucial for identifying salt-tolerant crops and protecting wetland ecosystems (Talke and Jay, 2020). Proper salinity will promote the growth of salt marsh plants, but exceeding the tolerance threshold will inhibit the growth of salt marsh plants to a certain extent (Warren, 2002; Eller et al., 2014), and even cause plant death and ecological degradation. High salinity stress may cause cell death and even plant mortality, but it has been demonstrated that reactive oxygen species (ROS) are key players in these processes of cell death (Distéfano et al., 2021). Membrane damage brought on by glutathione-dependent peroxidase inactivation characterizes ferroptosis, an iron-dependent type of regulated cell death (Dixon et al., 2012; Dixon and Stockwell, 2014; Stockwell et al., 2017). Iron, a vital element in plant aerobic respiration and energy metabolism, was analyzed due to its significant influence on plant physiological activity and nutrient absorption (Briat et al., 2007). Small compounds that inhibit glutathione peroxidase 4 (GPX4), a phospholipid peroxidase, result in the accumulation of ROS and induced ferroptosis cell death (Dixon et al., 2012; Yang et al., 2016). Researchers found that the tidal *P. australis* population was more tolerant to salt than the freshwater population, with numerous genes and alleles of antioxidant protection system enzymes related to salt damage (Holmes et al., 2016; Zhang et al., 2020), which laid the groundwork for future transgenic engineering and molecular marker development in salt-tolerant plants. However, despite extensive research on the salt tolerance mechanism of *P. australis* in coastal salt marshes (Gao et al., 2012; Schenck et al., 2018; Lambertini et al., 2020; Liu et al., 2021; Wu et al., 2022; Liu et al., 2023), there has still been a lack of research focused on the survival strategies and gene expression patterns of *P. australis* populations along tidal flat gradients in coastal marsh wetlands.

After suffering from the effects of tidal disturbance, the *P. australis* population in the Dongtan marsh wetland presented significant phenotypic differences at varying tidal flats, thus, providing a fascinating rationale to explore its survival strategies and salinity tolerance mechanisms. The *P. australis* populations at the middle and high tidal flats in the Dongtan marsh wetland were distributed in continuous bands and grew luxuriantly, while at the low tidal flat, it was distributed in fragmentary patches. Some populations in low tidal patches expanded annually and could flower, bear seeds, and complete their life history, while others gradually disappeared and could not complete their life history (unpublish data). The degradation of such a short-lived *P. australis* patch aggravated habitat fragmentation. These backgrounds underscored the need to explore the salt-tolerant survival strategies and mechanisms of the *P. australis* population in the different tidal flats. This study aimed to elucidate the survival strategies of *P. australis* at different tidal flats and the corresponding physiological mechanisms in the Dongtan salt marsh wetland. To this end, transcriptomes were utilized to identify DEGs across the *P. australis* habitats at different tidal flats, resulting in a set of candidate genes used to help unravel the basis of salinity tolerance in *P. australis*.

## Materials and methods

### Study area

The investigation was conducted in Dongtan, which is a coastal salt marsh wetland situated on Chongming island in the Yangtze River estuary (121°50′E-122°5′E, 31°25′N-31°38′N). The average annual temperature is 15.3°C, and the annual precipitation is 1100 mm. A dense network of tidal creeks characterizes the area; Dongtan wetland clearly exhibits zones of high, middle, and low tidal flats.

**Figure 1:**
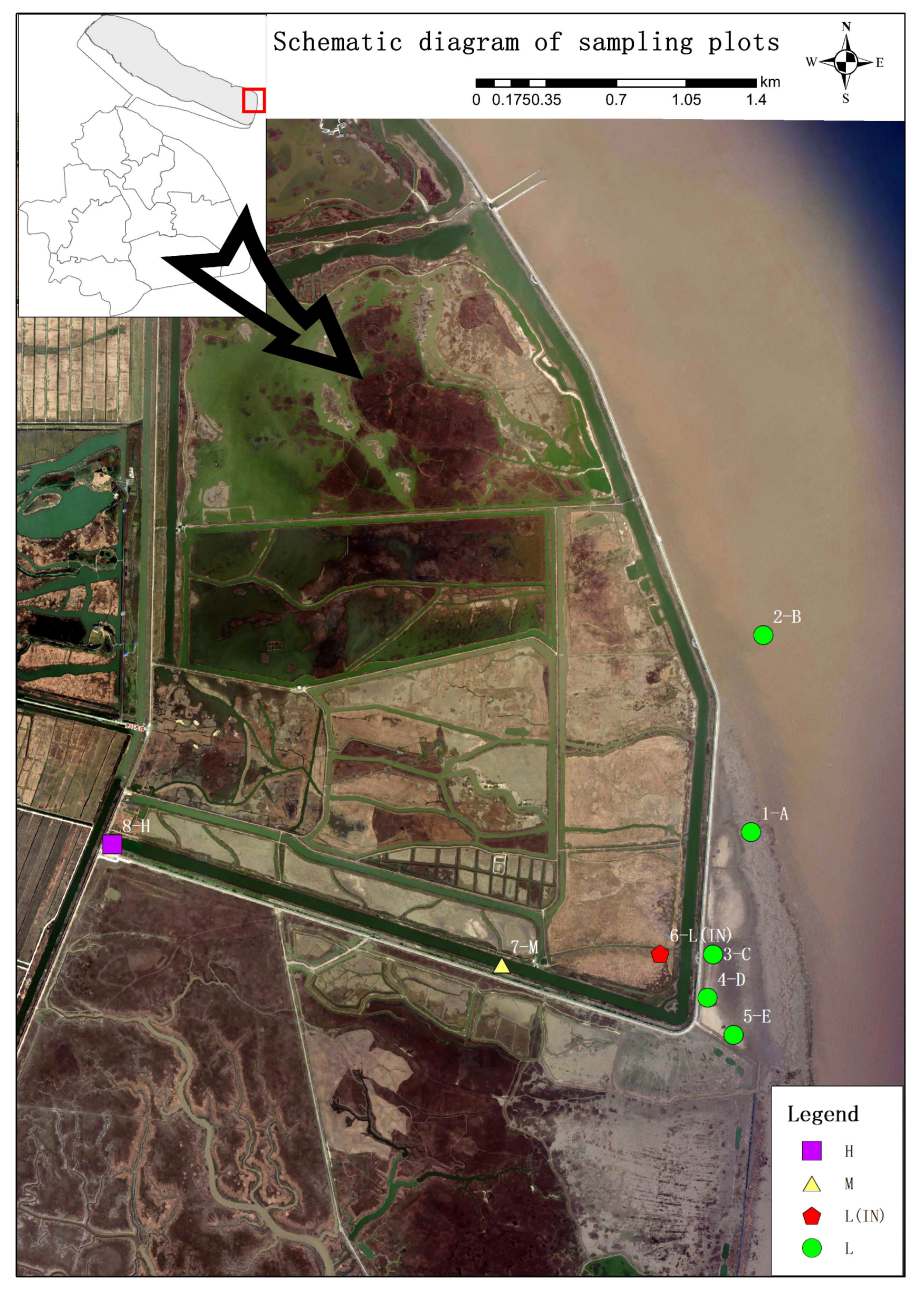
Study area and different sampling locations. H, high tidal flat; M, mid-tidal flat; L, low tidal flat outside the dike; L(IN), low tidal flat inside the dike.

Dongtan Wetland is located in the eastern part of Chongming Island, Shanghai, in the core location of the Yangtze River estuary, which is one of the largest and most typical estuarine tidal mudflat wetlands in China (**Figure 1**). The intertidal zone of the Dongtan wetland is host to a diverse array of plant species, including *Phragmites australis*, the dominant species of Poaceae, and *Scirpus mariqueter*, the endangered species of Cyperaceae, and some other plant species. The non-native species *Spartina alterniflora* was introduced to the area at the end of the 20^th^ century (Zuo et al., 2012), and it began to expand its distribution along the tidal flats, especially in habitats with relatively high soil salinity.

### Plant sampling and measurements

Sampling sites were set up in the habitats of the *P. australis* population at different tidal flats in the Dongtan salt marsh wetland. The sites near the coast were designated as the L, and those inside the dyke, built between 1998 and 2002, were designated as the IN. At the low tidal flat outside the dyke, 5 patches (A, B, C, D, and E) were randomly selected (the inter-patch distance was 500 m), and a 15 m × 15 m site was selected from each patch, and 3 sampling quadrats (1 m × 1 m) were randomly selected in the site. For sampling site inside the dyke (IN), 5 sampling quadrats (1 m × 1 m) were randomly set up at a distance of 50 m from each quadrat. A total of 10 sampling quadrats (1 m×1 m) were set up (the inter-patch distance was 20 m) at the middle (M) and high tidal flats (H). A total of 8 sites were systematically selected at the different tidal flats and total 40 soil samples were collected.

Five above-ground shoots of *P. australis* with uniform size were taken from each quadrat, and 40 × 5 = 200 above-ground plant samples were collected. The fresh plant weight (FW) was determined immediately after being returned to the laboratory, and the plants samples were stored properly and kept fresh for other physiological and biochemical analyses. Leaves for determination of leaf dry matter content and specific leaf area and plant dry weight samples were oven-dried at 80°C for 3 - 5 days to obtain a constant dry weight (DW).

For each shoot collected, we measured the biomass, shoot height, diameter, leaf tissue water content (LDMC), SPAD, and specific leaf area (SLA). The shoot’s height and diameter were measured using a tape measure and vernier caliper, respectively. Three fully unfolded green leaves were taken from each shoot, leaf area (LA) and dry weight (LDW) were measured. Use the ImageJ to calculate the leaf area. The specific leaf area is calculated by the formula: SLA=LA/LDW , and the tissue water content was calculated using the formula: LDMC=1−(FW−DW)/DW×100% . The formula for the coefficient of variation is as follows: $CV=\frac{\sigma }{\mu }\times 100\%$ , **σ** stands for standard deviation and μ represents the mean value.

### Soil physical and chemical properties

In each plot, three soil column samples (Φ = 4 cm, *H* = 10 cm) were randomly selected, and each soil column sample was divided into two depths (0–10 cm, 10–20 cm). Total Nitrogen (TN) was measured using the Kjeldahl method (Bremner, 1960). Total phosphorus (TP) was measured after digestion using HClO_4_–H_2_SO_4_ (Sommers and Nelson, 1972). Other physical and chemical properties of the soil samples, such as salinity, pH, available phosphorus (AVIP), nitrate-nitrogen (NO_3_^–^N), ammonium-nitrogen (NH_4_^+^-N), soluble organic carbon (SOC) were measured according to the methods described in Tuo et al. (Tuo et al., 2023).

### Iron content in plant leaves

Leaf samples (0.2 g, accurate to 0.0001 g) were weighed and placed in a microwave digestion tank. A total volume of 8 mL 65% nitric acid and 2 mL 35% hydrogen peroxide were added successively, and microwave digestion was carried out after adding zeolite. After cooling to room temperature, the sample solution in the digestion tank was rinsed twice with ultra-pure water, and the resulting wash solution was combined in a 50 mL volumetric flask with a constant volume of ultra-pure water up to a total volume of 50 mL, mixed and shaken sufficiently. A volume of 10 mL of the above sample solution was removed into four 50 mL volumetric flasks, to which the following volumes of iron standard solution were added: 0.0, 0.5, 1.0, and 2.0 mL. These solutions were diluted with water to the scale and shaken well before measurement. According to the working conditions selected when using inductively coupled plasma (ICP), a standard curve was established on the machine first, and the linear fitting degree reached a standard (*r* ≥ 0.999), then the samples were measured.

### Enzyme activity assay

To explore how the activity of antioxidant enzymes in leaves varies between different tide levels, the SOD, CAT, and POD enzyme activity were tested. The activities of CAT, POD and GPX were measured according to the method described by Tian et al. with a slight modification (Tian et al., 2016). The reaction mixture comprised 1.5 mL of 50 mmol/L phosphoric acid buffer (pH 7.0) (containing 0.1 mmol/L EDTA), 0.3 mL of 50 mmol/L guaiacol, 0.1 mL of 2% H_2_O_2_, and 0.1 mL enzyme solution. All reaction liquids were mixed evenly and quickly to initiate the reaction, which was scanned at 470 nm. Values were read every minute and repeated in triplicate. An increase of 0.01 per minute at OD470 was used as the activity unit to measure enzyme content.

The reaction mixture consisted of 0.2 mL of enzyme solution (the blank tube was replaced after heated boiling of inactivated enzyme solution), 1.5 mL of 50 nmol/L phosphate buffer (pH 7.0, containing 0.1 nmol/L EDTA), and 1.0 mL of distilled water. Prior to CAT determination, the reaction mixture was preheated with 0.1 mol/L of hydrogen peroxide in a 26 °C water bath. After preheating the reaction mixture, 0.3 mL hydrogen peroxide solution was added, mixed evenly, placed in an enzyme activator with a 240 nm wavelength, and the OD240 value was determined. CAT activity was estimated using the decrease in H_2_O_2_ based on the absorbance at 240 nm.

SOD activity was estimated by recording the enzyme-induced reduction in absorbance of the superoxide nitroblue tetrazole (NBT) complex (Giannopolitis and Ries, 1977). The reaction mixture (2 mL) contained 0.1 mL 150 mmol methionine, 0.1 mL 0.75M NBT, 0.1 mL 0.1m EDTA, 1.0 mL 100 mm potassium phosphate buffer, 0.5 mL distilled water, 0.1 mL 20 µM/L riboflavin and 0.1 mL enzyme extract. Each sample was tested in triplicate. Two tubes of enzyme-free extract served as background controls; one was left in the dark to serve as a blank. The other tubes, with or without enzyme solution, were placed under a 5000 Lux light source for 15-20 minutes. After photo-induction of the reaction, the tubes were placed in the dark to stop the reaction. Absorbance values were recorded at 560 nm. The inhibition of half of the photochemical reduction of NBT was considered one unit of enzyme activity.

GPX levels were examined by mixing the supernatant with the reaction cocktail (pH 7.0), including GPX assay buffer, DMSO (Beyotime, S0057S, China), 10 mM reduced glutathione solution, and 15 mM peroxide solution (t-Bu-OOH). The pH of the solution was adjusted to 7.0. The reaction mixture was mixed with 5,5′-dithiobis-(2-nitrobenzoic acid) (Beyotime, S0057S, China) before incubating in the dark for 10 min. Last, the mixed reaction compound was plated in triplicate, and the absorbance was measured at 412 nm.

### RNA extraction

A total of 48 leaf samples were selected from four sampling locations (L, IN, M, H) based on the tide level and analyzed using transcriptome sequencing. The total RNA of samples was isolated and purified using Trizol reagent (Invitrogen, Carlsbad, CA, USA) following the manufacturer’s procedure. Each sample’s RNA amount and purity were quantified using NanoDrop 2000 (NanoDrop, Wilmington, DE, USA). RNA integrity was assessed using an Agilent 2100 Bioanalyzer (Agilent Technologies, Palo Alto, CA) with an RIN of >7.0.

### Construction of RNA-seq libraries and sequencing

Total RNA was used for RNA-seq libraries construction: mRNA was enriched from the total RNA using oligo(dT)magnetic beads. Enriched RNAs were fragmented into small pieces using divalent cations under 85°C. The cleaved RNA fragments were reverse-transcribed to create cDNA, which was used to synthesize U-labeled second-stranded DNAs with *E. coli* DNA polymerase I RNase H and dUTP. An A-base was added to the blunt ends of each strand, preparing them for ligation to the indexed adapters. Each adapter contained a T-base overhang for ligating the adapter to the A-tailed fragmented DNA. Single or dual-index adapters were ligated to the fragments, and size selection was performed using AMPure XP beads. After the heat-labile UDG enzyme treatment of the U-labeled second-stranded DNA strands, the ligated products were amplified using PCR under the following conditions: initial denaturation at 98°C for 30 min; 14 cycles of denaturation at 98°C for 15 sec, annealing at 60°C for 30 sec, and extension at 72°C for 30 sec; and then a final extension at 72°C for 5 min. The average insert size of the cDNA library was 350 bp ( ± 50 bp). Last, sequencing (PE150 model) was performed on a GenoLab M (GeneMind Bio, China) following the vendor’s recommended protocol.

### Bioinformatics analysis

After removing samples with poor biological duplication, a total of 39 samples met the analysis criteria. First, Cutadapt (https://github.com/marcelm/cutadapt) (Zhang et al., 2021c) was used to remove reads containing adaptor contamination and low-quality or undetermined bases. The sequence quality was verified using FastQC (http://www.bioinformatics.babraham.ac.uk/projects/fastqc/). Hisat2 (Mann et al., 2022) was used to map reads to the *P. australis* genome. The mapped reads of each sample were assembled using StringTie (http://ccb.jhu.edu/software/stringtie/). Then, the transcriptomes from all samples were merged to reconstruct a comprehensive transcriptome using Perl scripts. After the final transcriptome was generated, StringTie (Ji et al., 2023) and Ballgown (Leek, 2014) were used to estimate all transcript expression levels. Simply put, it is used to determine mRNA expression levels by calculating the fragments per kilobase of transcript per million mapped reads (FPKM). The differentially expressed mRNAs were selected using a log2 (fold change) >1 or log2 (fold change)< -1, with statistical significance (FDR< 0.05) using the R package edgeR (Robinson et al., 2010). The hub genes of modules were identified, and correlations were utilized between modules and genes, as well as between genes and phenotype, to conduct screening (gene and module > 0.8; gene and phenotype > 0.5). Traditional singular enrichment analysis (SEA) was used for the enrichment analysis of GO terms and pathways. The enrichment P-value calculation was performed using Fisher’s exact test. A WGCNA was performed using averaged FPKM values and the WGCNA package in the R software program, based on the tutorial available on the WGCNA official website (https://labs.genetics.ucla.edu/horvath/CoexpressionNetwork/Rpackages/WGCNA/) (Langfelder and Horvath, 2008). Pearson correlation coefficient analysis was applied for the correlation analysis. The pheatmap package was used to draw the correlation heatmap. WGCNA and DEG analyses were used to explore the association between the specific core genes with degradation of the specific vegetation patch.

### Data analysis

Excel 2019, Origin 8, and SPSS 22.0 were used for data statistics and processing. Duncan’s method was used to compare mean values (*P<* 0.05).

Principal component analysis (PCA) was used to synthesize the functional traits of *P. australis* and the property variables of leaf antioxidant enzyme activity at each tidal flat. Redundancy analysis (RDA) was used to analyze the driving force factors of soil physicochemical properties on the functional traits of *P. australis* populations at different tidal flats. The “vegan” package was used for PCA and RDA; the “ggplot2” package and software Origin 2018 was used for plotting figures. Pearson correlation coefficient analysis was used for correlation analysis and fuzzy comprehensive evaluation by SPSS 22.0. We used plant fresh weight, dry weight, plant height, base diameter, LDMC, SLA and SPAD to calculate fuzzy comprehensive membership functions. The membership function value of the method is calculated as follows:

Synthesis of the membership function value formula (Equation 1):


(1) $$X_{i}\left(\mu \right)=\left[\sum X_{ij}\left(\mu \right)\right]/n$$

Establishment of the positive index membership function value formula (Equation 2):


(2) $$X_{xj}\left(\mu \right)=\left(X_{ij}-X_{jmin}\right)/\left(X_{jmax}-X_{jmin}\right)$$

Establishment of the negative index membership function value formula (Equation 3):


(3) $$X_{xj}\left(\mu \right)=1-\left(X_{ij}-X_{jmin}\right)/\left(X_{jmax}-X_{jmin}\right)$$

In the formula, $\sum X_{ij}\left(\mu \right)$ represents the cumulative membership function value of the j index of the *i*th variety; $X_{jmin}$ represents the minimum value of the *j*th index; $X_{jmax}\;$ represents the maximum value of the *j*th index; n represents the sample number. The comprehensive index was extracted from the single index using principal component analysis. The weight of each comprehensive index is found in Equation 4.


(4) $$W_{i}=\frac{P}{\sum_{i=1}^{n}P_{i}}\left(i=1,2,\ldots ,n\right)$$

where Wi represents the importance degree (weight) of the *i*th comprehensive indicator among all comprehensive indicators; Pi represents the contribution rate of the *i*th comprehensive index.

The integrated assessment value for salt tolerance (D) is defined in Equation 5:


(5) $$\sum_{i=1}^{n}\left[\mu \left(X_{i}\right)\times W_{i}\right]\left(i=1,2,\ldots ,n\right)$$

The membership function and comprehensive coefficients were used to investigate the comprehensive evaluation value of salt tolerance (D value) of *P. australis* functional traits. The membership function value of each comprehensive index was calculated using Formulas (2) and (3), the weight of each comprehensive index was calculated using Formula (4), and the comprehensive evaluation value of salt tolerance of each *P. australis* population (D value) was calculated using Formula (5).

## Results

### Growth characteristics of the *P. australis* population

**Figure 2:**
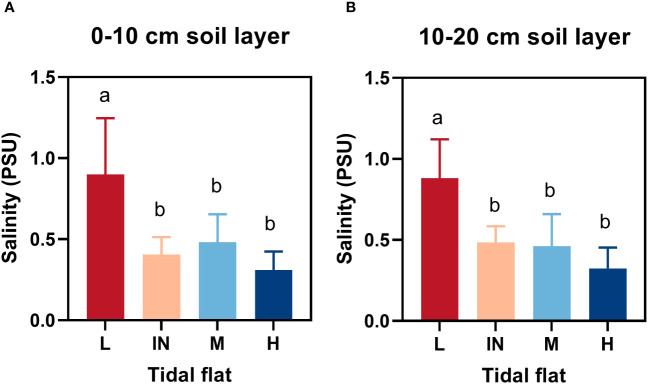
Soil salinity at different tidal flats. **(A)** and **(B)** represented 0-10cm and 10-20cm soil layers respectively. L, low tidal flat outside the dike; IN, low tidal flat inside the dike; M, mid-tidal flat; H, high tidal flat. Letters indicate significant groupings of soil salinity at different tidal flats from LSD test (*P* ≤ 0.05). Mean ± SD, n = 15(L), n = 5(IN), n = 10 (M and H, respectively).

There were significant differences in plant height, base diameter, fresh weight, dry weight, specific leaf area and leaf dry matter content among different tidal flats (*P*< 0.05). However, the relative content of chlorophyll (SPAD) did not differ in different tidal flats (**Table 1**). The soil salinity at low tidal flat was the highest in *P. australis* habitats at different tidal flats (**Figure 2**; **Supplementary Table S1**). The plant height varied from 108.93 to 199.56 cm at different tidal flats, and the coefficient of variation was 37.05% and 27.22%, respectively (**Supplementary Table S2**). When comparing low tidal and high tidal flats, the dry weight of the population decreased by 47.74% (**Table 1**). The plant weight of the *P. australis* communities in various tidal flats showed a significant coefficient of variation, both in fresh and dry weight (**Supplementary Table S2**). The basal diameter of the *P. australis* community was the largest at the high tidal flat (7.41 mm) and the smallest at the low tidal flat (IN) (4.99 mm) (**Table 1**).

**Table 1:** Results of one-way ANOVA for the effects of tidal flats on growth and physiological parameters of *Phragmites australis* community.

	L	L(IN)	M	H	F	p
Height (cm)	108.93 ± 40.16c	115.20 ± 17.27c	145.32 ± 34.51b	199.56 ± 54.33a	52.766	<0.001
BD (mm)	6.09 ± 1.34a	4.99 ± 1.20b	5.73 ± 1.39b	7.41 ± 1.98a	17.443	<0.001
FW (g)	33.19 ± 13.69b	26.57 ± 9.47b	35.47 ± 13.27b	65.54 ± 33.78a	33.585	<0.001
DW (g)	17.94 ± 6.95b	16.22 ± 5.35b	20.68 ± 7.03b	34.33 ± 15.31a	34.885	<0.001
SLA (cm^2^/g)	120.48 ± 24.60	131.77 ± 21.84	110.20 ± 19.93	110.76 ± 73.45	2.041	0.107
SPAD	44.12 ± 4.11	43.87 ± 3.89	43.29 ± 5.44	40.95 ± 2.1	1.294	0.291
LDMC (%)	0.44 ± 0.06bc	0.46 ± 0.04ab	0.49 ± 0.08a	0.51 ± 0.18a	7.924	0.002

BD, base diameter of *P. australis*; DW, total aboveground biomass; FW, total fresh aboveground weight; SPAD, total chlorophyll concentration; SLA, specific leaf area. Data was presented with mean ± SD. Letters indicate significant groupings from LSD post hoc tests (*P* ≤ 0.05).

**Figure 3:**
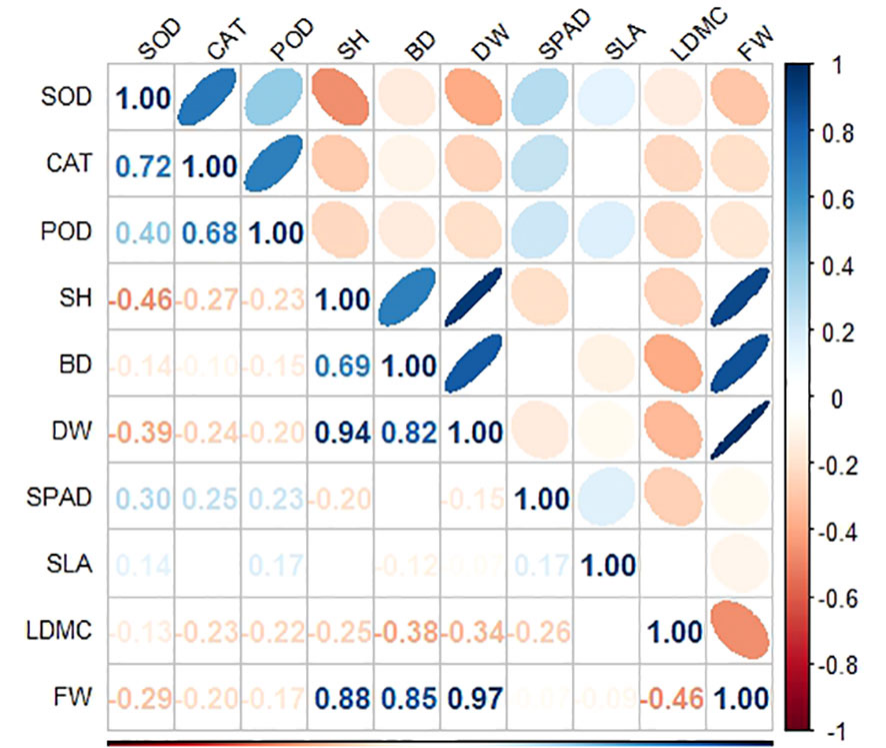
Correlation analysis of functional traits and antioxidant enzyme activity of *P. australis* under different tidal flats.

At the middle and high tidal flats, *P. australis* populations exhibited a higher allocation of resources towards biomass and plant height, with no significant difference in leaf traits between tidal levels. (**Table 1**). The shoot heights of different *P. australis* populations were significantly (*P<* 0.01) correlated with the biomass of vegetative organs in the above-ground part of the plant (**Supplementary Table S3**). LDMC were positively correlated to FW and DW but negatively correlated with BD (**Figure 3**; **Supplementary Table S3**).

### Principal component analysis of growth traits and a comprehensive evaluation value of plant membership function among *P. australis* resources under tidal disturbance

**Figure 4:**
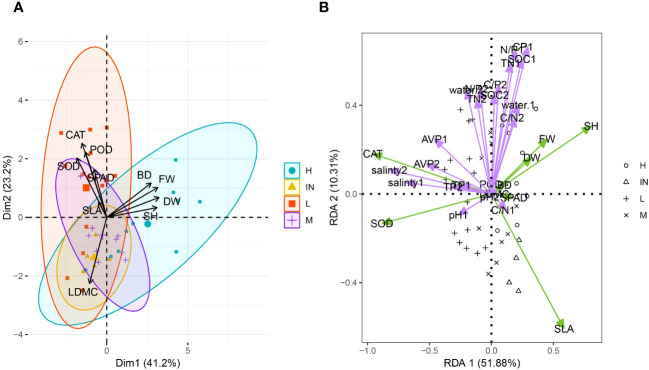
Principal component analysis of functional traits **(A)** and redundancy analysis of plant functional traits and soil physical and chemical properties **(B)** of *P. australis* at different tidal flats. SH, shoot height; BD, base diameter of *P. australis*; DW, total aboveground dry weight; FW, total aboveground fresh weight; SPAD, relative chlorophyll content; LDMC, leaf dry matter content; SLA, specific leaf area; POD, peroxidase; CAT, catalase; SOD, superoxide dismutase; AVP, Available phosphorus; TN, Total nitrogen; TP, Total phosphorus; C/N, Carbon nitrogen ratio; C/P, Carbon phosphorus ratio; N/P, Nitrogen phosphorus ratio. The number after different physical and chemical properties of soil represented different soil layers, 1 mean 0-10 cm soil layer and 2 mean 10-20 cm soil layer.

In principal component Axis 1, the average load values of plant height (SH), the shoot fresh weight (FW), the shoot dry weight (DW) and base diameter had higher component contribution rates. These four indexes positively affected the principal component Axis 1, indicating that plant height and biomass contribute most to the evaluation of the functional traits in *P. australis*. The contribution rate of the second principal component was 23.23%, and the eigenvalue was 2.32 (**Supplementary Table S4**). The eigenvectors of SOD, CAT, and POD, which had higher loads, also had positive effects (**Figure 4A**). These three physiological indexes could also be considered important evaluation indices for the stress resistance of populus *P. australis*.

In coastal salt-marsh wetland environments, soil salinity is an important limiting factor for plant survival. The salinity of soil at low tidal flat was significantly higher than that at middle and high tidal flat, and there was no difference between 0 - 10cm and 10 - 20cm soil layers (**Figure 2**). The membership function and comprehensive coefficients were used to investigate the salt resistance value (*D* value) of *P. australis* functional traits. Through comparison, it was found that Community B had the highest *D* value (0.7625) and the strongest salt tolerance. The *D* value of the E community was 0.1477, and the salt tolerance was weak (**Table 2**).

**Table 2:** Comprehensive index, membership function value, comprehensive evaluation value of *P. australis* at eight sampling sites.

Sample	Model evaluation				Comprehensive evaluation value D	Rank
	X1	X2	μ(X1)	μ(X2)		
A	0.1618	1.4151	0.2955	0.7407	0.6394	5
B	-0.6902	1.4009	0.1397	0.7384	0.7625	1
C	-0.2894	-0.1665	0.2130	0.4822	0.7476	2
D	0.0949	-0.5906	0.2833	0.4129	0.6977	4
E	-1.1151	0.8619	0.0619	0.6503	0.1477	8
IN	-0.5604	-0.3483	0.1634	0.4524	0.2056	7
M	-0.1571	-0.4364	0.2372	0.4381	0.7334	3
H	0.9887	-0.2656	0.4469	0.4660	0.4497	6

### Functional traits and soil physicochemical properties

The redundancy analysis (RDA) was performed to examine the driving force behind the growth characteristics among diverse plant community habitats. These results showed that the cumulative variance contribution rate of axis 1 and axis 2 in RDA was 61.19% (**Figure 4B**). The plant biomass, plant height, and basal diameter were most correlated with the water content, organic matter content, C/P, N/P, and TN content in the 0 - 10 cm soil layer. Soil salinity was negatively correlated with shoot height, plant biomass and SLA. SLA had the greatest correlation with the C/N in the first layer of soil. SPAD was also limited by soil moisture content and nutrients (TN, C/P, and P/N).

### Antioxidant enzymes in the leaves of *P. australis* populations at different tide levels

**Figure 5:**
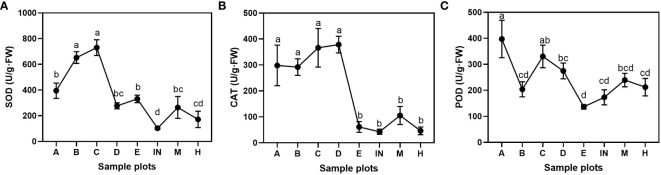
Antioxidant enzyme activities of *P*. *australis* leaves at different tidal flats. **(A)** superoxide dismutase activity; **(B)** catalase activity; **(C)** peroxidase activity. Letters indicate significant groupings from LSD *post hoc* tests (P ≤ 0.05). Mean ± SD, n = 3.

There were significant differences in peroxidase among *P. australis* populations at different tidal flats (*P*< 0.05). The superoxide dismutase and catalase were significantly different between *P. australis* populations (*P*< 0.01). The SOD activity of the *P. australis* leaves differed significantly in various places, and the order of enzyme activity was C ≥ B > A ≥ E ≥ D ≥ M ≥ H ≥ IN (**Figure 5A**). It was found that the rank of the CAT enzyme activity was similar to SOD activity in *P. australis* leaves, namely, D ≥ C ≥ A ≥ B > M ≥ E ≥ H ≥ IN (**Figure 5B**). The POD enzyme activity order was A ≥ C ≥ D ≥ M ≥ H ≥ B ≥ IN > E (**Figure 5C**).

### Identification of salt tolerance-associated genes

The salt tolerance of different groups is significantly different; this mechanism was explored using transcriptome sequencing of *P. australis*. After filtering out genes with an FPKM< 20 in all samples, 57,163 genes were obtained for WGCNA. The software’s power beta was set to 14, identifying 49 modules (**Supplementary Figure S1A**). Correlation analysis was performed on these modules based on soil salt concentrations in the 0 – 10 cm and 10 – 20 cm soil layers. Candidate modules that satisfied both the 0 – 10 cm and 10 – 20 cm soil salt concentration requirements with correlations greater than 0.5 were identified (**Supplementary Figure S1B**). After candidate screening, three modules (brown, dark, and darkorange2) were selected. The intersection of the two sets in three modules was 1070 hub genes related to salt tolerance (**Supplementary Figure S1C**). GO and pathway enrichment analysis of hub genes found that terms related to endoplasmic and stress response were significantly enriched, indicating that these genes may play a role in regulating the salt tolerance mechanism of *Phragmites australis* (**Supplementary Figure S1D**).

**Figure 6:**
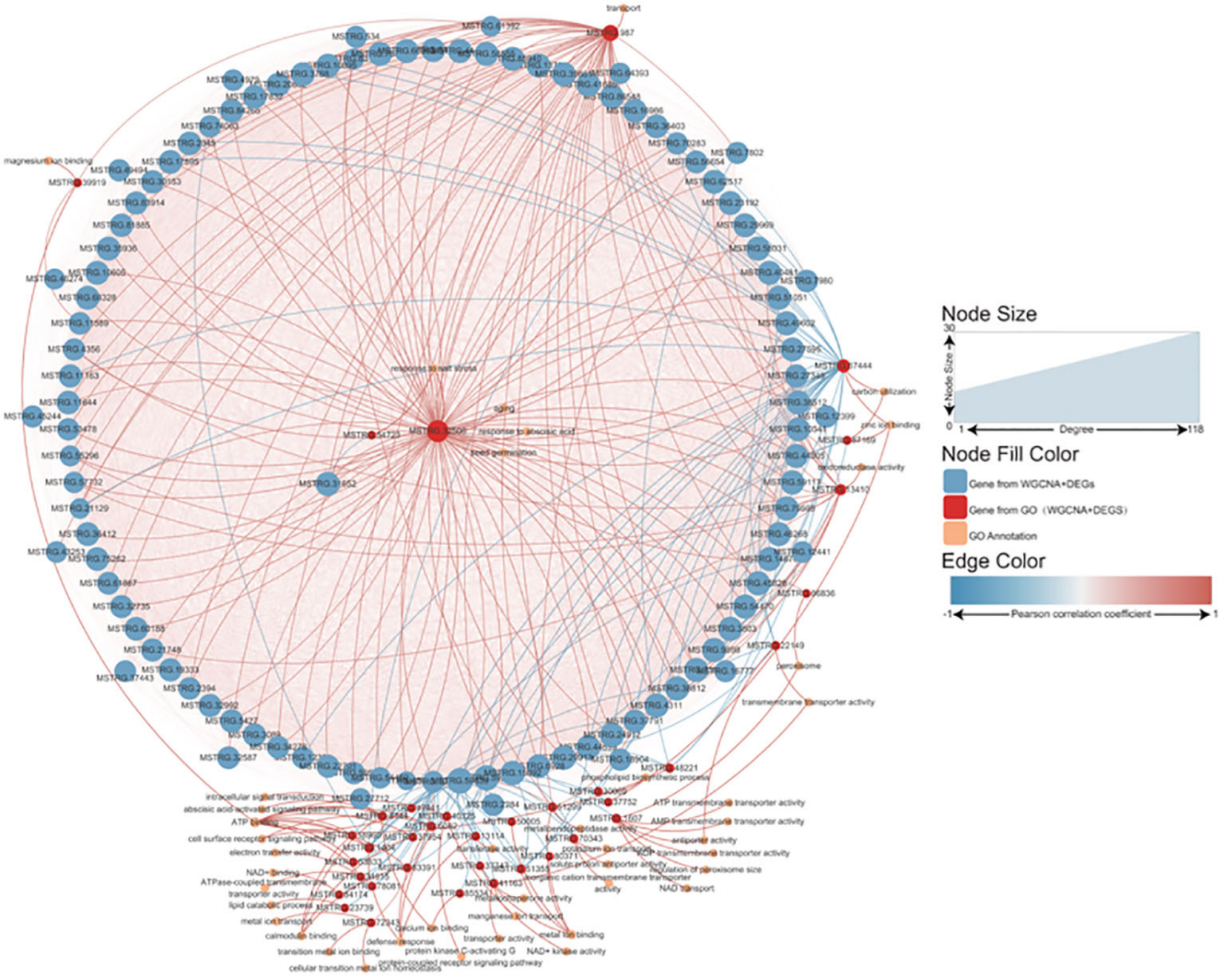
Network analysis of the salt tolerance genes. The thickness and thinness of the lines represented the high and low correlations, respectively, the red lines represented the positive correlation, and the blue lines represented the negative correlation. The blue circle represented salt tolerance genes from WGCNA and DEG, and the red circle represented the gene having GO annotation information. The orange circle represented the gene having GO annotation information that was not screened by WGCNA and DEG.

The L was used as the control to decipher the detailed information on salt-tolerance-related genes by comparing it to the other three tidal flats. This comparison revealed that the H exhibited the greatest number of DEGs, while the M showed the most similarity to the L (**Supplementary Figure S2A**). Among the three comparisons, 519 up-regulated and 712 down-regulated genes were found to be common among them (**Supplementary Figures S2B, C**). Combined gene sets created using WGCNA and DEG analysis found that 105 genes were shared (**Supplementary Figure S2D**). Interestingly, most of the 105 genes were highly expressed in the L. Unfortunately, the majority of these genes are unannotated in the database. A Pearson correlation coefficient network was constructed to explore the relationship between these genes and the *P. australis* salt tolerance mechanism (**Figure 6**). It revealed that 87 out of 105 genes were directly correlated with the gene *MSTRG.32506*, a gene involved in response to salt stress. In addition, most of the genes showed a positive correlation with each other. Typically, these data illustrated a strong correlation between the salt tolerance of *P. australis* and the expression of these 87 specific genes. Subsequently, GO enrichment analysis revealed some terms related to glutathione and iron ion binding rather than salt tolerance (**Supplementary Figure S3**).

### Identification of plant growth and degeneration-associated genes

**Figure 7:**
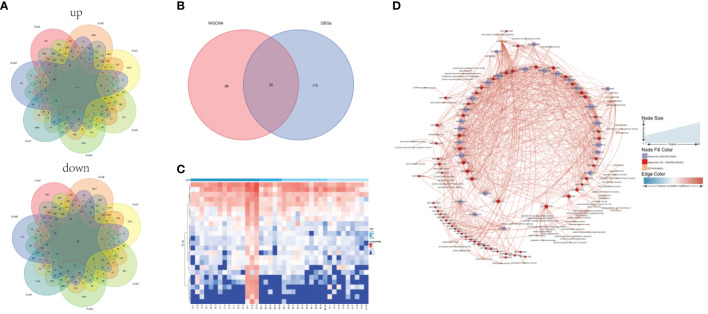
Screening plant growth and degeneration core gene set by DEG and WGCNA analysis. **(A)** The common intersection of 8 groups under the DEGs analysis method (compare with group E). **(B)** The common intersection of growth and degeneration genes under the DEGs and WGCNA analysis method. **(C)** The heat map showed the expression of the 25 shared genes. **(D)** Network analysis of the growth and degeneration genes. The thickness and thinness of the lines represented the high and low correlations, respectively, the red lines represented the positive correlation, and the blue lines represented the negative correlation. The blue circle represented growth and degeneration genes from WGCNA and DEG, and the red circle represented the gene having GO annotation information. The orange circle represented the gene having GO annotation information that was not screened by WGCNA and DEG.

**Figure 8:**
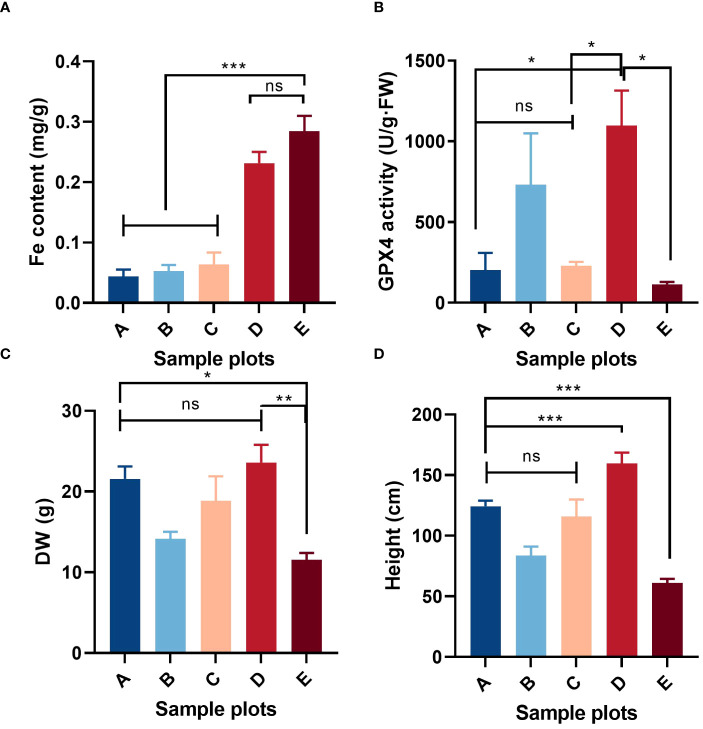
Situation of ferroptosis-related factors in the shoot of *P. australis* in **(E)** at low tidal flat: iron content, **(A)**; GPX enzyme activity, **(B)**; above ground dry weight, **(C)**; shoot height, **(D)**. Mean ± SD, n = 5. Significant Duncan test differences: *< 0.05, **< 0.01, ***< 0.001. ns (not significant).

Most vegetation indicators such as biomass, coverage, structure and species diversity changed when the patch vegetation degraded (Bardgett et al., 2021), which associated with the specific core genes. First, three gene modules were identified (white, dark turquoise, and pale turquoise) as significantly correlated with growth degeneration (**Supplementary Figure S1B**). Then, Patch E was used as a control and compared with the other seven sites resulting in the identification of seven DEG sets (**Supplementary Figure S2**). Upon intersecting these sets, 117 up-regulated and 83 down-regulated genes were obtained (**Figure 7A**). In combination with WGCNA, 25 common genes were identified (**Figure 7B**). Notably, all 25 genes were highly expressed in Patch E (**Figure 7C**), but they lacked annotation in the database. A correlation network was constructed to explore how these genes affect plant growth and degeneration. Interestingly, it revealed a significant association between the genes and ferroptosis-related terms, such as iron binding and cellular response to iron ion starvation (**Figure 7D**). Based on these findings; it was hypothesized that the degeneration of Patch E might be associated with ferroptosis. To validate it, the iron ion contents in the leaves of plants in Patch E were analyzed, and it was discovered that the content of Fe^3+^ in Patch E was not significantly reduced (**Figure 8A**). The GPX4 enzyme activity was significantly lower in Patch E compared to the other groups (**Figure 8B**).

**Figure 9:**
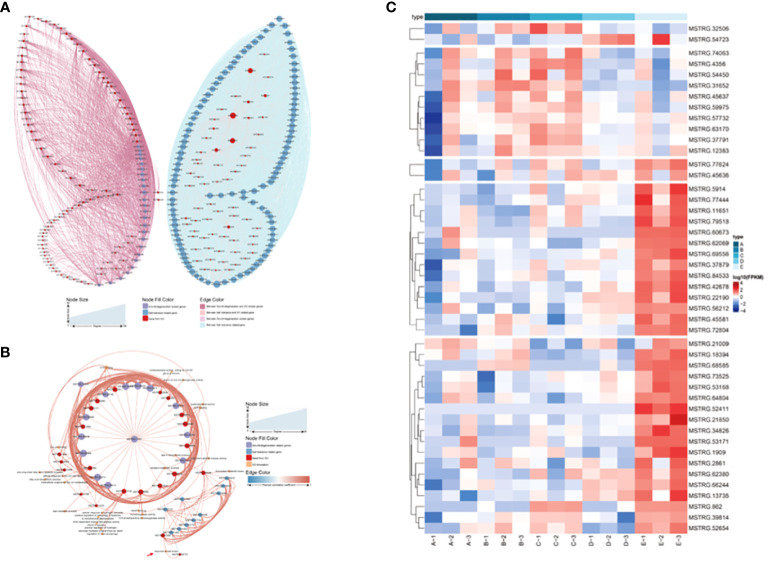
Network analysis of the growth & degeneration with salt tolerance genes. **(A)** Network analysis of the growth & degeneration with salt tolerance genes. **(B)** Network analysis of the ferroptosis with salt tolerance genes. **(C)** The heat map showing the expression of the shared genes in network analysis of the ferroptosis with salt tolerance genes.

Patch E is a particular *P. australis* population that tolerates salt, growing in the low tidal flat, experiencing ferroptosis, and falling into degeneration. It spurs greater interest in understanding the association between salt tolerance and ferroptosis. A correlation network was constructed with 105 genes associated with salt tolerance and 25 genes related to growth degradation (**Figure 9A**). MSTRG.45636 and MSTRG.77624 act as the central links in the complex network of correlations. To further enhance the understanding of the cross-link between ferroptosis and degeneration, genes related to ferroptosis and salt tolerance were chosen to reconstruct the network. It revealed that *MSTRG32506* and *MSTRG37791* are the crucial nodes in the network, and *MSTRG.11651* could be a potential core gene (**Figure 9B**). Furthermore, these genes were highly expressed in Patch E (**Figure 9C**). Co-expression indicated the association between salt tolerance and ferroptosis in the *P. australis*.

## Discussion

### Effects of environmental variables on functional traits of *P. australis*

Wetlands around the world are dynamic ecosystems particularly vulnerable to biological invasion. Native species have strong adaptability and resistance to environmental changes such as pests and disasters (Peng et al., 2018). *P. australis* is an essential native species, highly adaptable to various environments in coastal wetland ecosystems. *P. australis* absorbs nutrients and heavy metals, builds and stabilizes soil, and creates self-sustaining vegetation in urban and industrial areas where many other plants do not flourish (Eller et al., 2020).

*P. australis* can survive not only in freshwater habitats, but also in salt marsh environments. In coastal wetlands, *P. australis* requires a certain amount of salt for vegetative growth, especially when it grows to the heading to flowering stage. Appropriate salt supplementation can increase fiber content and biomass, making it healthier and more robust (Guan et al., 2017). Soil salinity plays a dominant role in determining the growth of *P. australis*, and high salinity limits the population’s survival (Schenck et al., 2018; Jiao et al., 2020; Zhang et al., 2021b; Barbafieri et al., 2023). The results showed that the variation coefficients of plant height at low tidal flat and biomass at high tidal flat of *P. australis* were 37.06% and 44.6%, respectively, indicating that these two morphological traits were largely affected by environmental factors (**Table 2**). Soil water content, pH and salinity all affected the spatial distribution intensity of *P. australis*. Specifically, the spatial distribution pattern intensity was positively correlated with density, height, biomass and stem diameter (*P*< 0.01) (Jiao et al., 2020). Soil salinity plays a leading role in the stoichiometric characteristics of carbon, nitrogen, and phosphorus in *P. australis* leaves and roots (Zhang et al., 2021b), which indicates that soil salinity also indirectly influences the stoichiometric characteristics of soil carbon, nitrogen, and phosphorus in *P. australis* habitat. Our results also showed that the C:N:P stoichiometric pattern of soil was significantly positively correlated with plant height, fresh weight, dry weight and base diameter of *P. australis* (**Figure 4B**). Soil salinity plays a leading role in *P. australis* leaf traits and enzyme activity (**Figure 4B**). Soil salinity at low tidal flat severely limits the plant height and biomass of common reed population (**Table 2**). Previous studies have suggested that the salt tolerance of this species may be related to phylogenetic differentiation (Gao et al., 2012; Lambertini et al., 2020; Liu et al., 2021). Gao et al. also suggested that the correlation between phenotype and environment may be reflected at the level of individual genetic polymorphisms (Gao et al., 2012). Haplotype P is the dominant species of *P. australis* in eastern China, and studies have shown that haplotype P (octoploid) has longer, wider and thicker leaves than haplotype O (tetraploid), and the leaf dry mass and stem biomass of haplotype P are significantly higher than that of haplotype O, as well as the stem diameter (Liu et al., 2021). Consequently, the evolutionary difference of polyploidy and soil salinity may have a significant association with the varying ecological performance of the common reed population in the salinity heterogeneity in coastal wetlands. Recent studies have determined that the successful restoration of *P. australis* populations in the Chongming Wetland at the Yangtze Estuary is regulated by both water level and degree of salinization (Wang et al., 2006). In the same greenhouse setting, *P. australis* seedlings from various origins exhibited changes in morphology and growth across salinity gradients, showing that the observed phenotypic diversity was genetic (Ren et al., 2020; Wu et al., 2022). Regardless of the difference, increased pore water salinity consistently lowered common reed stem density, height, and biomass in studies (Schenck et al., 2018). Appropriate salinity can promote photosynthesis, and an appropriate water level is necessary for population recovery (Zhou et al., 2015). The SPAD value can be used to determine the actual nitrate demand of plants to guide the addition of nitrogen fertilizer to soil. In disturbed environments, SLA also has different degree of intraspecific variation (Schneider et al., 2017), regulated by chemicals in leaves and restricted by soil factors.

Physiological traits included enzyme activity and chlorophyll content. A previous study has shown that soil traits are more likely to influence them than morphological traits (Zhang et al., 2020). Enzyme activity refers to the life activity of an organism, showing the order of its internal chemical reaction process. Many factors regulate this order, leading to metabolic disorders, disease, and even death once out of it. Plants that live in salty, flooded wetlands for a long time accumulate large amounts of reactive oxygen species (ROS) in their bodies. ROS scavengers in the body’s antioxidant protection enzyme system mainly include GPX, CAT, POD, SOD, etc (Apel and Hirt, 2004). SOD converts superoxide into hydrogen peroxide through a disproportionation reaction, and APX and GPX are responsible for removing hydrogen peroxide (Apel and Hirt, 2004). SOD is the forerunner of the O_2_^-^ scavenging reaction in cells. It first converts harmful O_2_^-^ into H_2_O_2_, and then CAT and POD in the body help to decompose H_2_O_2_ into H_2_O and O_2_ (Tian et al., 2016). These antioxidant enzyme protection systems function together to resist adversity. The results of this study showed that the activities of SOD, CAT, POD, and GPX in *P. australis* populations at the low tidal flat were higher than at the middle and high tidal flat; still, the enzyme activities were also divergent among different patch populations, which may be related to the heterogeneity of habitats (**Figure 5**).

### Differential gene expression related to salt tolerance

Salt stress hinders the growth and development of above-ground tissues, and salt’s regulation of shoot apical meristem and shoot architecture is poorly understood (Aslam et al., 2011). Although the molecular mechanisms in roots have been extensively studied, it cannot be deduced that the same is true for above-ground tissues (Zhao et al., 2013; Shen et al., 2018a; Li et al., 2023). A new study has revealed that the signaling pathways of brassinosteroids and abscisic acid are involved in reducing far red (shade)-induced hypocotyl elongation caused by low soil NaCl levels (Tan et al., 2018; Hayes et al., 2019). Research has demonstrated that plants employ diverse coping mechanisms to manage salt stress (Ali and Yun, 2017; Hernández, 2019; Hurtado et al., 2020). For instance, the tetraploid black locust resists salt stress by increasing chromosome ploidy (Wang et al., 2013). Some species apply the secretion of salt and/or the succulence of plant organs as a tolerance mechanism to high salt concentrations (Behr et al., 2017). Additionally, *Arabidopsis* adjusts its flowering time when subjected to salt stress (Kim et al., 2007; Zheng et al., 2022). This study corroborates these findings regarding plants’ strategies for managing salt stress. In this study, the authors delved deeper into the salt tolerance mechanism of the common *P. australis* using transcriptome sequencing and WGCNA. A core set of genes related to salt tolerance was discovered, which can serve as a valuable reference for future research on the salt tolerance of the common *P. australis*. For instance, Gene *MSTRG.32506* and gene *MSTRG.54723* were not only annotated by the GO database as responsive to salt stress but were also identified as key players in the network analysis of salt tolerance genes (**Figure 6**, **Figure 9**). However, the lack of annotation for most genes in the database has posed challenges in further understanding their functions. Therefore, functional experiments are planned to investigate the role of these genes in the *P. australis* response to salt stress.

### Ferroptosis-like cell death in *P. australis*

Ferroptosis is a form of cell death dependent on iron and is non-apoptotic (Dixon, 2017). It is characterized by mitochondrial shrinkage, accumulation of iron and lipid ROS (Lewerenz et al., 2018), depletion of glutathione, decreased activity of GPX4 (Conlon and Dixon, 2017; Seibt et al., 2019), and aggravation of lipid peroxidation damage (Ursini and Maiorino, 2020). This form of cell death has also been observed in plants and was first discovered in *Arabidopsis thaliana* root cells under heat stress. Later, it was reported as a mechanism in rice resistant to *Magnaporthe oryzae* (Dangol et al., 2019; Shen et al., 2020; Liang et al., 2021; Sánchez-Sanuy et al., 2022). Ferroptosis-like cell death has also been observed in prokaryotes under heat stress, indicating that this may be an ancient cell death process that is conserved in both eukaryotes and prokaryotes (Distéfano et al., 2017; Shen et al., 2018b; Kazan and Kalaipandian, 2019; Xie et al., 2021). However, salt stress-induced ferroptosis in plant cells has not been widely reported. Some studies have confirmed that the limitation of iron intake can reduce the ROS emergency response in plants (Witzel et al., 2009). The growth and degeneration of *P. australis* growth indices are essential indicators of salt tolerance and adaptability. Compared with the other patches at low tidal flat, the growth indices of patch E were poor and showed a tendency toward degeneration (**Figure 8**; **Supplementary Table S5**). This study provides evidence that salt stress can cause iron death in plants by acquiring evidence of ferroptosis occurring in Patch E under salt stress, as indicated by increased Fe concentration and decreased GPX4 activity. The presence of iron ions in Patch E confirmed the functionality of the GPX4 enzyme, which is known to play a role in ferroptosis (**Figures 7**, **8**). These results indicated that ferroptosis might play a key role in the degeneration of *P. australis* growth areas (Stockwell et al., 2017; Bardgett et al., 2021). However, additional experiments, such as transmission electron microscopy, could not be performed, and the current results only support the occurrence of ferroptosis under plant salt stress.

In summary, this study provided a promising direction for further research into the molecular mechanisms of plant cells coping with salt stress, and the relationship between ferroptosis and salt stress warrants further investigation.

## Data availability statement

The data presented in the study are deposited in the https://db.cngb.org/cnsa repository repository, accession number CNP0004592.

## Ethics statement

Animal experiments were not involved in this study, so animal ethical review is not applicable in this study.

## Author contributions

JJ: Data curation, Investigation, Software, Writing – original draft. XCZ: Software, Writing – original draft. PJ: Data curation, Writing – review & editing. XZ: Software, Writing – review & editing. DL: Writing – review & editing. YL: Writing – review & editing. LH: Writing – review & editing.

## Supplementary material

Supplementary Figure 1 The WGCNA analysis of all samples and identification of candidate hub genes. **(A)** The clustered modules of WGCNA. **(B)** Composite expected modules screened by WGCNA. **(C)**The Veen plot showed the interaction between 0-10cm and 10-20cm in three modules. **(D)** GO and KEGG enrichment analysis of the hub genes.

Supplementary Figure 2 Screening salt tolerance genes by DEG analysis. **(A)** Differential expression analysis results for the four tidal flats (compare with L). At each tidal flat, the total number of up-regulated (orange) and down-regulated (blue) genes was shown as a histogram. **(B)** The common intersection of up-regulated genes under the low, high, low (IN), and middle tidal flat. **(C)** The common intersection of down-regulated genes under the low, high, low (IN), and middle tidal flat. **(D)** The common intersection of salt tolerance genes under the DEGs and WGCNA analysis method.

Supplementary Figure 3 Analysis of GO biological processes (GO-BP), GO molecular functions (GO-MF) and GO cell composition (GO-CC) in the bubble chart.

Supplementary Figure 4 Screening growth & degradation genes by DEG Analysis. **(A)** Differential expression analysis results for the 8 sites (compared with E site). At each site, the total number of up-regulated (blue) and down-regulated (orange) genes were shown as a histogram.
