# Supplementary material for: Ecophysiological responses of Phragmites australis populations to a tidal flat gradient in the Yangtze River Estuary, China
Source: Front Plant Sci. 2024 Apr 30;15:1326345. doi: 10.3389/fpls.2024.1326345 (PMC11097105; doi:10.3389/fpls.2024.1326345)
Supplement: Supplementary file 1 [file DataSheet_1.docx]

TABLE S1 Results of two-way ANOVAs testing the effects of soil depth, the tidal flat and their interaction on the soil physicochemical properties under reed communities at different plots.

| factor | Soil layer | Tidal flat | | | | F | | |
| --- | --- | --- | --- | --- | --- | --- | --- | --- |
|  |  | L | L(IN) | M | H | T | S | T ⅹ S |
| Water contont(%) | 0-10cm | 0.46±0.09b | 0.31±0.03a | 0.35 ±0.08b | 0.56±0.24a | 12.080*** | 2.260 | 1.815 |
|  | 10-20cm | 0.47±0.07a | 0.34 ±0.06ab | 0.27 ±0.05bc | 0.43 ±0.17a |  |  |  |
| pH | 0-10cm | 8.46 ±0.43ab | 8.16 ±0.20bc | 8.56 ±0.34a | 8.27 ±0.18ab | 5.428** | 4.496* | .591 |
|  | 10-20cm | 8.49 ±0.33b | 8.29 ±0.47b | 8.83 ±0.35a | 8.49 ±0.17b |  |  |  |
| Salinity(psu) | 0-10cm | 0.90 ±0.35a | 0.41 ±0.11b | 0.48 ±0.17b | 0.31 ±0.12bc | 32.562*** | .067 | .153 |
|  | 10-20cm | 0.89 ±0.24a | 0.49 ±0.09b | 0.46 ±0.19b | 0.32 ±0.13b |  |  |  |
| AVP(mg/kg) | 0-10cm | 21.05±3.08a | 13.46±4.85b | 10.37±2.63c | 14.58±5.49b | 31.003*** | 3.371 | .196 |
|  | 10-20cm | 19.76±3.52a | 12.06±6.49b | 8.74±2.46b | 11.55±5.54b |  |  |  |
| TN(%) | 0-10cm | 0.09 ±0.01a | 0.07 ±0.01b | 0.05 ±0.02b | 0.11 ±0.06a | 8.292*** | 1.756 | .638 |
|  | 10-20cm | 0.09 ±0.02a | 0.06 ±0.03ab | 0.05 ±0.01bc | 0.08 ±0.05a |  |  |  |
| TP | 0-10cm | 0.06 ±0.02a | 0.06 ±0.01a | 0.06 ±0.002b | 0.06 ±0.01b | 17.056*** | .530 | .235 |
|  | 10-20cm | 0.06 ±0.02a | 0.06 ±0.002a | 0.05 ±0.003b | 0.057±0.007b |  |  |  |
| Organic carbon(%) | 0-10cm | 0.80 ±0.118a | 0.58 ±0.146b | 0.58 ±0.172b | 1.04 ±0.58a | 7.644*** | 2.011 | .607 |
|  | 10-20cm | 0.81 ±0.165a | 0.52 ±0.314a | 0.42 ±0.126ab | 0.81 ±0.556a |  |  |  |
| C/N | 0-10cm | 8.46 ±0.923ns | 8.01 ±0.723ns | 10.52±3.84ns | 9.50 ±1.427ns | 1.869ns | 2.373ns | 2.273ns |
|  | 10-20cm | 8.34 ±0.518ns | 8.34 ±1.966ns | 8.00 ±0.813ns | 9.12 ±2.221ns |  |  |  |
| C/P | 0-10cm | 12.60±1.80a | 9.40 ±2.70b | 10.27±3.09b | 16.96±7.48a | 8.602*** | 2.772 | .720 |
|  | 10-20cm | 12.65±2.417a | 8.28 ±4.84ab | 7.56 ±2.108bc | 13.57±7.84a |  |  |  |
| N/P | 0-10cm | 1.49 ±0.196a | 1.16 ±0.274b | 1.05 ±0.353b | 1.78 ±0.808a | 8.705*** | 2.382 | .734 |
|  | 10-20cm | 1.51 ±0.270a | 0.94 ±0.392b | 0.93 ±0.204b | 1.42 ±0.756a |  |  |  |

Notes：AVP, Available phosphorus; TN, Total nitrogen; TP, Total phosphorus; C/N, Carbon nitrogen ratio; C/P, Carbon phosphorus ratio; N/P, nitrogen phosphorus ratio. Values are presented as means ± SD. ** and *** represents significant difference between the different group at 0.01 and 0.001level, respectively. Different lowercase letters indicate significant differences at 0.05 level.

TABLE S2 Results of coefficient of variation of functional traits of different tidal flat *P.australis* population

|  | L | L(IN) | M | H | F | *p* |
| --- | --- | --- | --- | --- | --- | --- |
| Height(cm) | 37.06% | 14.98% | 23.75% | 27.22% | 52.766 | <0.001 |
| Base  diameter(mm) | 22% | 24.05% | 24.26% | 26.72% | 17.443 | <0.001 |
| Fresh  weight(g) | 41.25% | 35.64% | 37.38% | 51.53% | 33.585 | <0.001 |
| Total dry biomass(g) | 38.8% | 32.92% | 33.98% | 44.6% | 34.885 | <0.001 |
| SLA(cm^2^/g) | 20.41% | 16.57% | 18.09% | 66.31% | 2.041 | <0.001 |
| SPAD | 9.3% | 8.8% | 12.56% | 5.1% | 1.294 | 0.291 |
| LDMC(%) | 13.24% | 8.39% | 16.16% | 16.42% | 7.924 | <0.001 |

TABLE S3 Correlation analysis of 10 physiological characters in common reed

| variable | SOD | CAT | POD | SH | BD | DW | SPAD | SLA | LDMC | FW |
| --- | --- | --- | --- | --- | --- | --- | --- | --- | --- | --- |
| SOD | 1 | 0.718  *** | 0.397  *** | -0.463  *** | -0.143 | -0.386  *** | 0.297 | 0.144 | -0.135 | -0.292 |
| CAT | 0.718  *** | 1 | 0.683  *** | -0.272 | -0.104 | -0.244 | 0.254 | 0.039 | -0.230 | -0.203 |
| POD | 0.397  *** | 0.683  *** | 1 | -0.227 | -0.149 | -0.204 | 0.229 | 0.171 | -0.222 | -0.166 |
| SH | -0.463  *** | -0.272 | -0.227 | 1 | 0.685*** | 0.940  *** | -0.204 | -0.003 | -0.250 | 0.884  *** |
| BD | -0.143 | -0.104 | -0.149 | 0.685  *** | 1 | 0.820  *** | -0.015 | -0.115 | -0.381  *** | 0.852  *** |
| DW | -0.386  *** | -0.244 | -0.204 | 0.940  *** | 0.820  *** | 1 | -0.146 | -0.070 | -0.339  * | 0.968  *** |
| SPAD | 0.297 | 0.254 | 0.229 | -0.204 | -0.015 | -0.146 | 1 | 0.166 | -0.259 | -0.071 |
| SLA | 0.144 | 0.039 | 0.171 | -0.003 | -0.115 | -0.070 | 0.166 | 1 | 0.031 | -0.095 |
| LDMC | -0.135 | -0.230 | -0.222 | -0.250 | -0.381  *** | -0.339* | -0.259 | 0.031 | 1 | -0.465  *** |
| FW | -0.292 | -0.203 | -0.166 | 0.884  *** | 0.852  *** | 0.968  *** | -0.071 | -0.095 | -0.465*** | 1 |

Notes：SH stands for plant height, BD represents base diameter, FW represents the total fresh weight of the above-ground part of the plant, DW represents the total dry weight of the above-ground part of the plant, SPAD represents the relative chlorophyll content, SLA represents the specific leaf area, LDMC represents the leaf dry matter content, SOD represents the superoxide dismutase, CAT represents the catalase, POD represents peroxisome.Values are presented as correlation coefficient. *,** and *** represents significant difference between the different group at 0.05,0.01 and 0.001level, respectively.

TABLE S4 Characteristic value, contribution rate and cumulative contribution rate of principal component analysis

| variable | Dim1 | Dim2 | Dim3 | Dim4 | Dim5 |
| --- | --- | --- | --- | --- | --- |
| SOD | -0.547242 | 0.600681 | -0.099529 | 0.053582 | 0.513328 |
| CAT | -0.451130 | 0.748992 | -0.234195 | 0.256446 | 0.089509 |
| POD | -0.388404 | 0.658418 | -0.023171 | 0.352540 | -0.435295 |
| SH | 0.9198312 | 0.098254 | 0.104433 | 0.188900 | -0.032198 |
| BD | 0.8118941 | 0.346064 | -0.066895 | -0.031901 | 0.258435 |
| DW | 0.956486 | 0.200706 | 0.030585 | 0.104639 | 0.036026 |
| SPAD | -0.229191 | 0.485211 | 0.342352 | -0.688895 | -0.004819 |
| SLA | -0.148276 | 0.154718 | 0.914592 | 0.286870 | 0.066422 |
| LDMC | -0.313778 | -0.668800 | 0.074018 | 0.297389 | 0.304682 |
| FW | 0.936113 | 0.302655 | -0.003013 | 0.012775 | 0.044768 |
| Eigenvalue | 4.123259 | 2.323279 | 1.040780 | 0.886045 | 0.629393 |
| Percentage of Variance (%) | 41.23 | 23.23 | 10.40 | 8.86 | 6.20 |
| Cumulative (%) | 41.23 | 64.46 | 74.87 | 83.73 | 90.02 |

Notes：SH stands for plant height, BD represents base diameter, FW represents the total fresh weight of the above-ground part of the plant, DW represents the total dry weight of the above-ground part of the plant, SPAD represents the relative chlorophyll content, SLA represents the specific leaf area, LDMC represents the leaf dry matter content, SOD represents the superoxide dismutase, CAT represents the catalase, POD represents peroxisome.

TABLE S5 Growth of *Phragmites australis* in different patches at low tidal flat. Letters indicate significant groupings from LSD post hoc tests (P ≤ 0.05).

|  | A | B | C | D | E | F | P |
| --- | --- | --- | --- | --- | --- | --- | --- |
| Height  (cm) | 124.27±8.08b | 83.74±12.82c | 115.93±24.20b | 159.60±15.49a | 61.13±5.59c | 19.913 | 0.000 |
| Biomass(g) | 21.57±2.68a | 14.14±1.53bc | 18.86±5.24ab | 23.58±3.80a | 11.54±1.47c | 7.086 | 0.006 |
| Density(shoot/m^2^) | 12.67±2.52b | 20.33±1.53a | 13.00±1.00b | 20.00±2.65a | 3.33±4.16c | 21.24 | 0.000 |
| Coverage (%) | 75 | 80 | 80 | 85 | 30 | - | - |
