# Supplementary material for: Ecophysiological responses of Phragmites australis populations to a tidal flat gradient in the Yangtze River Estuary, China
Source: Front Plant Sci. 2024 Apr 30;15:1326345. doi: 10.3389/fpls.2024.1326345 (PMC11097105; doi:10.3389/fpls.2024.1326345)
Supplement: Supplementary Figure 1 — The WGCNA analysis of all samples and identification of candidate hub genes. (A) The clustered modules of WGCNA. (B) Composite expected modules screened by WGCNA. (C)The Veen plot showed the interaction between 0-10cm and 10-20cm in three modules. (D) GO and KEGG enrichment analysis of the hub genes. [file DataSheet_2.docx]

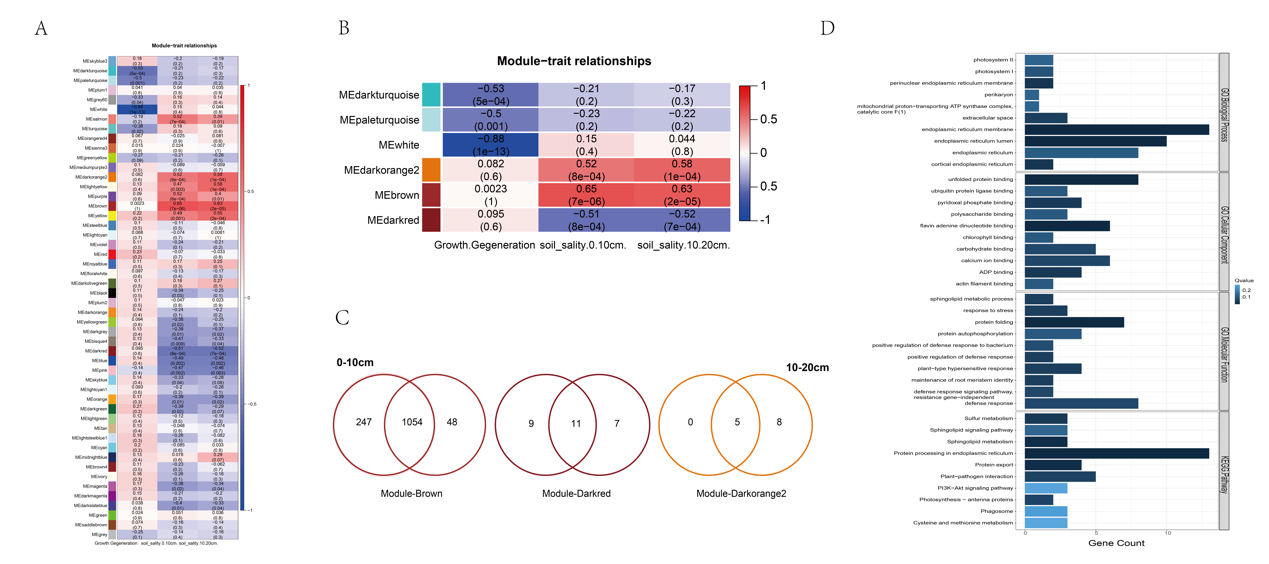


Figures S1: The WGCNA analysis of all samples and identification of candidate hub genes. (A) The clustered modules of WGCNA. (B) Composite expected modules screened by WGCNA. (C)The Veen plot showed the interaction between 0-10cm and 10-20cm in three modules. (D) GO and KEGG enrichment analysis of the hub genes.


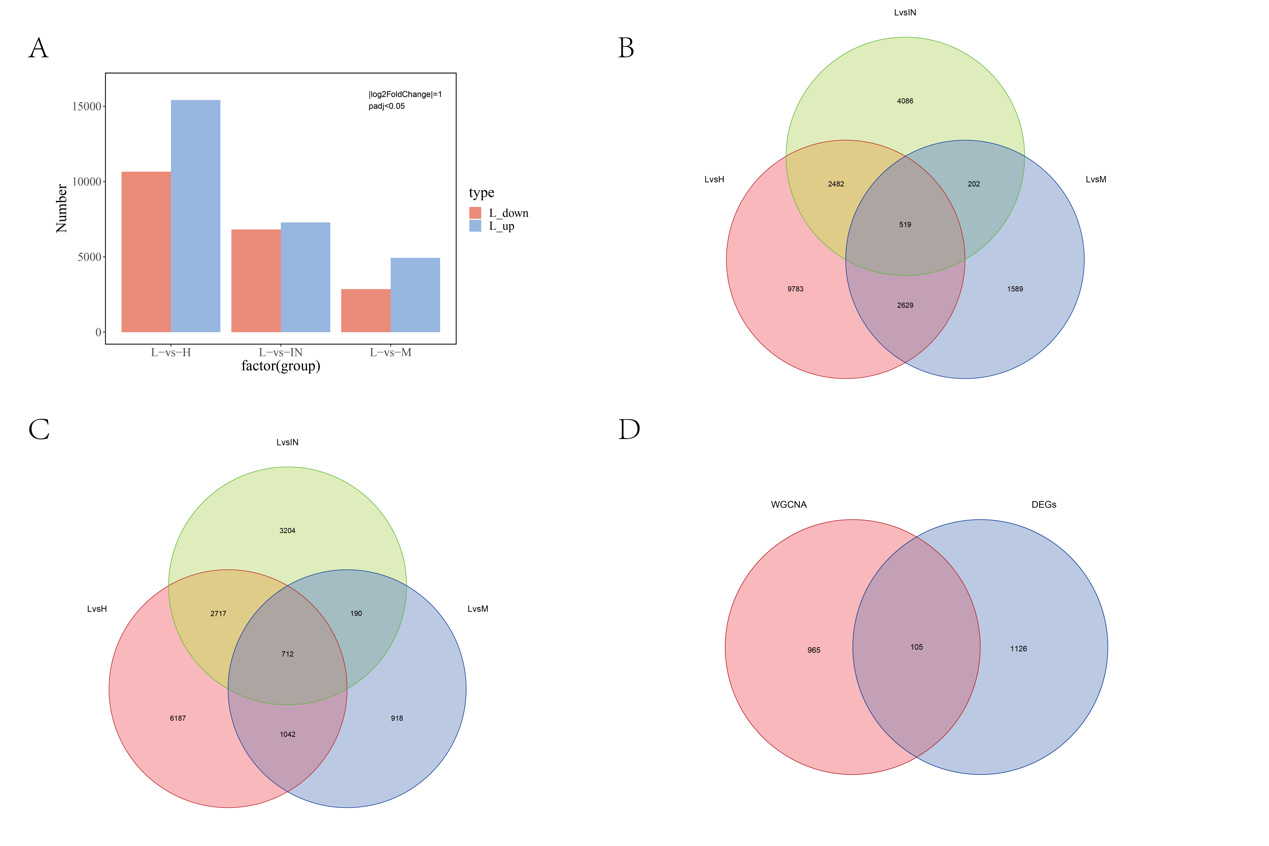


Figure S2: Screening salt tolerance genes by DEG analysis. (A) Differential expression analysis results for the four tidal flats (compare with L). At each tidal flat, the total number of up-regulated (orange) and down-regulated (blue) genes was shown as a histogram. (B) The common intersection of up-regulated genes under the low, high, low (IN), and middle tidal flat. (C) The common intersection of down-regulated genes under the low, high, low (IN), and middle tidal flat. (D) The common intersection of salt tolerance genes under the DEGs and WGCNA analysis method.

Figure S3: Analysis of GO biological processes (GO-BP), GO molecular functions (GO-MF) and GO cell composition (GO-CC) in the bubble chart.

Figure S4: Screening growth & degradation genes by DEG Analysis. (A) Differential expression analysis results for the 8 sites (compared with E site). At each site, the total number of up-regulated (blue) and down-regulated (orange) genes were shown as a histogram.
